# Virulence factors, serogroups and antimicrobial resistance properties of *Escherichia coli* strains in fermented dairy products

**DOI:** 10.1186/1756-0500-7-217

**Published:** 2014-04-07

**Authors:** Farhad Safarpoor Dehkordi, Farshad Yazdani, Jalal Mozafari, Yousef Valizadeh

**Affiliations:** 1 Young Researchers Club and Elite, ShahreKord Branch, Islamic Azad University, P.O. Box 166, ShahreKord, Iran

**Keywords:** Shiga toxin-producing *Escherichia coli*, Virulence factors, Antibiotic resistance properties, Fermented dairy products, Iran

## Abstract

**Background:**

From a clinical perspective, it is essential to know the microbial safety of fermented dairy products. Doogh and kashk are fermented dairies. These products are used by millions of people but their microbial qualities are unknown. Shiga toxin producing *Escherichia coli* (STEC) is one of the most commonly detected pathogens in the cases of food poisoning and food-borne illnesses. The present investigation was carried out in order to study the molecular characterization and antimicrobial resistance properties of STEC strains isolated from fermented dairy products.

**Methods:**

Six hundred fermented dairy samples were collected and immediately transferred to the laboratory. All samples were cultured immediately and those that were *E. coli*-positive were analyzed for the presence of O157 , O26, O103, O111, O145, O45, O91, O113, O121 and O128 STEC serogroups, *tetA*, *tetB*, *blaSHV*, *CITM*, *cmlA*, *cat1*, *aadA1*, *dfrA1*, *qnr*, *aac* (*3*)-*IV*, *sul1* and *ereA* antibiotic resistance genes and *stx1*, *stx2*, *eaeA*, *ehly*, *cnf1*, *cnf2*, *iutA*, *cdtB*, *papA*, *traT*, *sfaS* and *fyuA* virulence factors using PCR. Antimicrobial susceptibility testing was performed also using disk diffusion methodology with Mueller–Hinton agar.

**Results:**

Fifty out of 600 (8.33%) dairy samples harbored *E. coli*. In addition, yoghurt was the most commonly contaminated dairy. O157 (26%) and O26 (12%) were the most commonly detected serogroups. A significant difference was found between the frequency of Attaching and Effacing *E. coli* and Enterohaemorrhagic *E. coli* (*P* <0.05). *Stx1* (44%), *eae* (36%), *papA* (32%) *stx2* (30%), and *ehly* (28%) were the most commonly detected virulence factors. The genes encode resistance against tetracycline (*tetA* and *tetB*) (76% and 70%, respectively), cephalothin (*blaSHV*) (38%), ampicillin (*CITM*) (36%) and gentamicin (*aac* (*3*)-*IV*) (32%) were the most commonly detected. High resistance levels to tetracycline (84%), penicillin (46%), ampicillin (38%) and streptomycin (36%) were observed.

**Conclusion:**

Fermented dairy products can easily become contaminated by antibiotic resistant STEC strains. Our findings should raise awareness about antibiotic resistance in Iran. Clinicians should exercise caution when prescribing antibiotics, especially in veterinary treatments.

## Background

Dairy products are raised as complete foods especially for juveniles. Their high value for proteins, minerals, fats and vitamins is undeniable. Doogh (also known as yoghurt drink) is a yogurt-based beverage which is popular in Iran and also found in Afghanistan, Azerbaijan, Armenia, Iraq, Syria, Turkey, Pakistan and Balkans. Kashk is a thick whitish liquid similar to whey, used in traditional Persian cooking. Kashk is a fermented dairy product manufactured traditionally in dried form and produced industrially in liquid form in Iran. Dried kashk is a concentrated yogurt-type product produced with dehydration of homemade yogurt by sun-drying in summer months by nomads and villagers in the different regions of Iran [1]. Doogh, yoghurt and kashk are rich in potassium, calcium, protein and group B vitamins. In a day, Millions of people use from these dairy products in their routine meal. Unfortunately, adequate heat and time were not performed in their traditionally producing.

Shiga toxin-producing *Escherichia coli* (STEC) is one of the most common milk-borne pathogens [2-4]. Infection with STEC strains can result in a spectrum of outcomes, ranging from asymptomatic carriage to uncomplicated diarrhea, bloody diarrhea, hemolytic uremic syndrome (HUS), thrombocytopenia, hemolytic anemia, and acute renal failure [5-8]. High mortality and morbidity rates have been reported for HUS, which can occur from infection with STEC strains [5,9,10]. The pathogenesis of *E. coli* is related to several bacterial virulence factors [8,11,12]. Some of the most important virulence factors in *E. coli* strains are the intimin (*eae*) protein, two phage-encoded cytotoxins called *stx1* and *stx2*, the plasmid-encoded enterohemolysin or enterohaemorrhagic *E. coli* (EHEC) protein known as hemolysin (*ehly*) [8,11,12]. The Cytotoxic Necrotizing Factor (*cnf*) is another putative toxin which is responsible for induces enlargement and multinucleation of cultured eukaryotic cells [13].

A broad spectrum of Gram-negative bacterial species has been shown to produce Cytolethal Distending Toxin (CDT) [14], and three closely linked genes (*cdtA*, *cdtB*, and *cdtC*) are required for toxin expression [14].

Most outbreaks and sporadic cases of bloody and non-bloody diarrhea and HUS have been attributed to strains of the STEC serogroup O157 [15,16]. However, non-O157 strains such as O26, O103, O111, O145, O45, O91, O113, O121 and O128 have been shown to cause food poisoning, HUS, bloody diarrhea, and other gastrointestinal illnesses [2,15-17].

Diseases caused by *E. coli* often require antimicrobial therapy; however, antibiotic-resistant strains of this bacterium cause longer and more severe illnesses than their antibiotic-susceptible counterparts. Several studies have shown that antibiotic resistance in *E. coli* has increased over time [2,16,18-21]. In keeping with this, an epidemiological investigation in Iran revealed that STEC strains were the most commonly detected strains in patients with diarrhea and that there was a high incidence of resistance (85–100%) to commonly used antibiotics [22-25].

There were no data on the distribution of serogroups, virulence genes and the antimicrobial resistance properties of *E. coli* strains isolated from yoghurt, kashk and doogh. Therefore, the aim of the present study was to characterize *E. coli* strains isolated from yoghurt, kashk and doogh at the molecule level and investigate their susceptibility to 14 commonly used antibiotics.

## Methods

### Sampling and *Escherichia coli* identification

Overall 600 dairy products including 200 yoghurt, 200 doogh and 200 kashk samples were purchased from supermarkets and retailers in various parts of Iran at summer of 2012. All of these dairy products were made traditionally by native people and after collection were kept under refrigeration in plastic bags. Samples were transported under refrigeration (at 4-6°C) in thermal boxes containing ice packs. All samples were diluted in phosphate buffered saline (PBS, Merck, Germany). A 25 g portion of each sample was blended with 225 mL of nutrient broth (Merck, Germany) for 2 min, using a Stomacher lab blender and incubated at 37°C for 24 h. A 1 mL sample of the nutrient broth culture was mixed with 9 mL of MacConkey broth (Merck, Germany) and further incubated at 37°C for 24 h. One loop of each tube was streaked on MacConkey agar (Merck, Germany). Such colonies were confirmed as *E. coli* using standard biochemical tests (e.g., Indole, Methyl red, Voges-Proskauer and Citrate utilization tests). Colonies were confirmed as *E. coli* by PCR [26]. *E. coli* isolates were stored in Tryptic Soy Broth (TSB, Merck, Germany) containing 20% glycerol at -70°C for further characterization.

### Antimicrobial susceptibility testing

Antimicrobial susceptibility testing of the isolates was performed using the Kirby–Bauer disc diffusion method and Mueller–Hinton agar (Merck, Germany) according to Clinical and Laboratory Standards Institute (CLSI) guidelines [27]. Inoculated plates were incubated aerobically at 37°C for 18–24 h, after which antimicrobial susceptibility in the *E. coli* isolates were tested. Penicillin (10 μg/disk), tetracycline (30 μg/disk), streptomycin (10 μg/disk), chloramphenicol (30 μg/disk), sulfonamide (100 μg/disk), sulfamethoxazole (25 μg/disk), gentamicin (10 μg/disk), cephalothin (30 μg/disk), trimethoprim (5 μg/disk), enrofloxacin (5 μg/disk), ciprofloxacin (5 μg/disk), ampicillin (10 u/disk), and nitrofurantoin (300 μg/disk) were tested. The results were interpreted in accordance with CLSI criteria [27]. *E. coli* ATCC 25922 was used as quality control for antimicrobial susceptibility determination.

### DNA extraction

Bacterial strains were grown overnight in Trypticase Soy Agar (TSA, Merck, Germany) at 37°C. A single colony was suspended in 100 μL of sterile distilled water. After boiling the suspension for 13 min, the suspension was frozen and centrifuged at 14,000 rpm for 15 min to pellet the cell debris [28]. The supernatant was used as a template for PCR amplification.

### PCR detection of serogroups, virulence factors and antibiotic resistance genes in STEC strains

The PCR assays, specific primer sequences and the predicted size of the amplified products for the different pathogenic gene coding regions including, *cnf1*, *cnf2*, *stx1*, *stx2*, *eaeA*, *cdtB*, *papa*, *sfaS*, *fyuA*, *iutA*, *traT*, and *hlyA* were employed as previously described [29-33].

To detect serogroups and antibiotic resistance genes in the *E. coli* isolates, several PCR assays were used. The primer sequences are summarized in Table 1. A DNA thermo-cycler (Eppendorf Mastercycler, Eppendorf-Nethel-Hinz GmbH, Hamburg, Germany) was used in all PCR reactions. The amplified DNA products were electrophoresed on 2% agarose gels at 90 V for 3 h using 1× TBE (0.89 M Tris borate, 0.02 M EDTA, pH 8.3) as the running buffer, then stained with ethidium bromide (10 mg/ml). Gels were visualized using a UV gel documentation system (Uvitech, UK). DNAs of *E. coli* O157:K88ac:H19, CAPM 5933, O159:H20 and CAPM 6006 strains were used as positive controls and distilled water was used as a negative control.

**Table 1:** Primer sequence for detection of STEC serotgroups and antibiotic resistance genes

**Primer name**	**Sequence**	**Size of product (bp)**	**Reference**
O26-F	(F) CAGAATGGTTATGCTACTGT	423	[34]
O26-R	(R) CTTACATTTGTTTTCGGCATC		
O103-F	(F) TTGGAGCGTTAACTGGACCT	321	[34]
O103-R	(R) GCTCCCGAGCACGTATAAG		
O111-F	(F) TAGAGAAATTATCAAGTTAGTTCC	406	[34]
O111-R	(R) ATAGTTATGAACATCTTGTTTAGC		
O145-F	(F) CCATCAACAGATTTAGGAGTGT	609	[34]
O145-R	(R) TTCTACCGCGAATCTATC		
O157-F	(F) CGGACATCCATGTGATATGG	259	[34]
O157-R	(R) TTGCCTATGTACAGCTAATCC		
O45-F	(F) CCGGGTTTCGATTTGTGAAGGTTG	527	[35]
O45-R	(R) CACAACAGCCACTACTAGGCAGAA		
O91-F	(F) GCTGACCTTCATGATCTGTTGA	291	[36]
O91-R	(R) TAATTTAACCCGTAGAATCGCTGC		
O113-F	(F) GGGTTAGATGGAGCGCTATTGAGA	771	[37]
O113-R	(R) AGGTCACCCTCTGAATTATGGCAG		
O121-*F*	(F) TGGCTAGTGGCATTCTGATG	322	[38]
O121-*R*	(R) TGATACTTTAGCCGCCCTTG		
O128-F	(F) GCTTTCTGCCGATATTTGGC	289	[39]
O128-R	(R) CCGACGGACTGATGCCGGTGATT		
*aadA1*	(F) TATCCAGCTAAGCGCGAACT	58	[40]
	(R) ATTTGCCGACTACCTTGGTC		
*tetA*	(F) GGTTCACTCGAACGACGTCA	57	[40]
	(R) CTGTCCGACAAGTTGCATGA		
*tetB*	(F) CCTCAGCTTCTCAACGCGTG	56	[40]
	(R) GCACCTTGCTGATGACTCTT		
*dfrA1*	(F) GGAGTGCCAAAGGTGAACAGC	45	[41]
	(R) GAGGCGAAGTCTTGGGTAAAAAC		
*qnr*	(F) GGGTATGGATATTATTGATAAAG	50	[20]
	(R) CTAATCCGGCAGCACTATTTA		
*aac* (*3*)-*IV*	(F) CTTCAGGATGGCAAGTTGGT	55	[42]
	(R) TCATCTCGTTCTCCGCTCAT		
*Sul1*	(F) TTCGGCATTCTGAATCTCAC	47	[42]
	(R) ATGATCTAACCCTCGGTCTC		
*blaSHV*	(F) TCGCCTGTGTATTATCTCCC	52	[42]
	(R) CGCAGATAAATCACCACAATG		
*CITM*	(F) TGGCCAGAACTGACAGGCAAA	47	[42]
	(R) TTTCTCCTGAACGTGGCTGGC		
*ereA*	(F) GCCGGTGCTCATGAACTTGAG	52	[42]
	(R) CGACTCTATTCGATCAGAGGC		
*cat1*	(F) AGTTGCTCAATGTACCTATAACC	55	[42]
	(R) TTGTAATTCATTAAGCATTCTGCC		
*cmlA*	(F) CCGCCACGGTGTTGTTGTTATC	55	[42]
	(R) CACCTTGCCTGCCCATCATTAG		

### Statistical analyses

The data were analyzed using SPSS software (Version 17. SPSS Inc, United States) to find any significant correlation between incidences of virulence factors, serogroups and antibiotics resistance properties of *E. coli* strains isolated from dairy products. Statistical significance was regarded at a P value < 0.05.

## Results

All of the dairy samples were tested using culture and PCR techniques. Distribution of *E. coli* strains in yoghurt, doogh and kashk is shown in Table 2. From 600 dairy samples, 50 (8.33%) were positive for *E. coli*. We found that yoghurt were the most commonly contaminated dairy (10%). We also found that O157 (26%) and O26 (12%) were the most commonly detected STEC O-serogroups among dairy samples. Distribution of putative virulence genes among *E. coli* strains isolated from dairy samples is shown in Table 3. *Stx1* (44%) had the highest incidence, followed by *eae* (36%), *papA* (32%) and *stx2* (30%). The Attaching and Effacing *E. coli* (AEEC) subtype was most commonly detected subtype (Table 4). All of the EHEC strains of our study harbored all of the *stx1*, *eae* and *ehly* genes (Table 4).

**Table 2:** Prevalence of STEC serogroups isolated from fermented dairy products in Iran

**Samples**	***E. coli *** **positive (%)**	**O157 (%)**	**O145 (%)**	**O128 (%)**	**O121 (%)**	**O113 (%)**	**O111 (%)**	**O103 (%)**	**O91 (%)**	**O45 (%)**	**O26 (%)**	**Non detected (%)**
Yoghurt (200)	20 (10)	5 (25)	1 (5)	2 (10)	1 (5)	1 (5)	1 (5)	1 (5)	-	1 (5)	3 (15)	4 (20)
Doogh (200)	14 (7)	3 (21.42)	1 (7.14)	1 (7.14)	-	1 (7.14)	1 (7.14)	1 (7.14)	1 (7.14)	-	2 (14.28)	3 (21.42)
Kashk (200)	16 (8)	5 (31.25)	1 (6.25)	1 (6.25)	1 (6.25)	1 (6.25)	1 (6.25)	1 (6.25)	1 (6.25)	1 (6.25)	1 (6.25)	2 (12.5)
Total (600)	50 (8.33)	13 (26)	3 (6)	4 (8)	2 (4)	3 (6)	3 (6)	3 (6)	2 (4)	2 (4)	6 (12)	9 (18)

**Table 3:** Distribution of virulence genes in STEC strains isolated from fermented dairy products in Iran

**Positive samples**	**Virulence genes**											
	***stx1***	***stx2***	***eae***	***ehly***	***cnf1***	***cnf2***	***iutA***	***cdtB***	***pap A***	***tra T***	***sfaS***	***fyu A***
Yoghurt (20)	12	8	9	6	6	4	2	3	7	5	2	3
Doogh (14)	4	3	3	4	1	1	1	1	3	2	1	-
Kashk (16)	6	4	6	4	2	3	2	1	6	3	1	-
Total (50)	22 (44)	15 (30)	18 (36)	14 (28)	9 (18)	8 (16)	5 (10)	5 (10)	16 (32)	10 (20)	4 (8)	3 (6)

**Table 4:** Distribution of virulence genes STEC seorgoups isolated from fermented dairy products in Iran

**Serotype**		**No. positive samples (%)**	**Virulence gene (%)**
Non STEC		6 (12)	-
STEC (88%)	EHEC	13 (26)	*stx1*, *eae*, *ehly*: 13 (100)
	AEEC	31 (62)	*stx1*: 22 (70.96) *stx2*: 15 (48.38) *eae*: 18 (58.06) *stx1*, *eae*: 14 (45.16) *stx2*, *eae*: 10 (32.25) *stx1*, *stx2*, *eae*: 7 (22.58)

Dairy samples of the present study had the pH range of 4–6.1. The distribution of antimicrobial resistance genes within the STEC serogroups isolated from dairy samples is shown in Table 5. Genes that encode resistance to tetracycline, tetracycline, cephalothin, ampicillin and gentamicin antibiotics, i.e., *tetA* (76%), *tetB* (70%), *blaSHV* (38%), *CITM* (36%) and *aac* (*3*)-*IV* (32%) were the most common antibiotic resistance genes in STEC serogroups isolated from dairy samples. Antimicrobial resistance pattern in the STEC serogroup isolates from the dairy samples is shown in Table 6. STEC strains exhibited the highest level of resistance to tetracycline (84%), followed by penicillin (46%), cephalothin (42%), ampicillin (38%) and streptomycin (36%). We found that 50% of tested strains were resistant to more than one antibiotic.

**Table 5:** Distribution of antibiotic resistance genes in STEC serogroups isolated from fermented dairy products in Iran

**STEC serotypes**	**Antibiotic resistance genes**											
	***aadA1***	***tetA***	***tetB***	***dfrA1***	***qnr***	***aac *** **( *3 *)-*IV***	***Sul1***	***blaSHV***	***CITM***	***ereA***	***cat1***	***cmlA***
O157 (13)	4	9	11	1	1	5	2	6	6	4	3	3
O145 (3)	2	3	2	1	1	1	1	3	2	1	1	1
O128 (4)	1	4	4	-	1	2	1	1	1	1	1	1
O121 (2)	-	2	2	-	-	-	1	2	-	-	1	1
O113 (3)	-	2	1	-	-	-	-	-	-	-	-	-
O111 (3)	-	1	1	-	-	-	-	-	-	-	-	-
O103 (3)	-	1	1	-	-	-	-	-	-	-	-	-
O91 (2)	-	1	-	-	-	-	-	-	-	-	-	-
O45 (2)	-	2	1	-	-	-	-	-	1	-	-	-
O26 (6)	2	5	6	1	1	2	1	3	2	1	1	1
Non detected (9)	6	8	6	2	2	6	3	4	6	2	4	5
Total (50)	15 (30)	38 (76)	35 (70)	5 (10)	6 (12)	16 (32)	9 (18)	19 (38)	18 (36)	9 (18)	11 (22)	12 (24)

**Table 6:** Antibiotic resistance pattern in STEC serogroups isolated from fermented dairy products in Iran

**STES serotypes**	P10* (%)	**TE30 (%)**	**S10 (%)**	**C30 (%)**	**SXT (%)**	**GM10 (%)**	**E15 (%)**	**NFX5 (%)**	**L2 (%)**	**CF30 (%)**	**CIP5 (%)**	**TMP5 (%)**	**F/M300 (%)**	**AM10 (%)**
O157 (13)	5	10	5	3	3	4	3	2	5	5	2	2	3	4
O145 (3)	3	2	3	4	2	4	-	1	3	3	1	1	1	4
O128 (4)	3	4	2	1	-	1	1	-	2	2	1	-	1	1
O121 (2)	1	2	2	2	1	1	2	1	1	2	-	-	1	3
O113 (3)	-	1	-	-	-	-	-	-	-	-	-	-	-	-
O111 (3)	1	2	-	-	-	1	-	-	-	-	-	-	-	-
O103 (3)	-	1	-	-	-	-	-	-	-	-	-	-	-	-
O91 (2)	-	1	-	-	-	-	-	-	-	-	-	-	-	-
O45 (2)	1	2	-	-	-	-	-	-	-	1	-	-	-	1
O26 (6)	3	4	2	2	1	2	1	1	3	2	-	-	2	1
Non detected (9)	6	13	4	3	3	4	3	3	3	6	3	3	3	5
Total (50)	23 (46)	42 (84)	18 (36)	15 (30)	10 (20)	17 (34)	10 (20)	8 (16)	17 (34)	21 (42)	7 (14)	6 (12)	11 (22)	19 (38)

*In this table P10 = penicillin (10 μg/disk); *TE*30 = tetracycline (30 μg/disk); S10 = streptomycin (10 μg/disk); C30 = chloramphenicol (30 μg/disk); *SXT* = sulfamethoxazol (25 μg/disk); *GM*10 = gentamycin (10 μg/disk); *E*15 = erythromycin (15 μg/disk); NFX5 = enrofloxacin (5 μg/disk); *L*2 = lincomycin (2 μg/disk); *CF*30 = cephalothin (30 μg/disk); *CIP*5 = ciprofloxacin (5 μg/disk); TMP5 = trimethoprim (5 μg/disk); F/M300 = nitrofurantoin (300 μg/disk); *AM*10 = ampicillin (10 u/disk).

## Discussion

Our work has identified the high levels of contamination in yoghurt, doogh and kashk with *E. coli* strains. The distribution of *E. coli* strains in yoghurt, doogh and kashk were 10%, 7% and 8%, respectively. There were significant differences (*P* <0.05) in the incidence of *E. coli* strains between the yoghurt and doogh. Of the studies that have been conducted in this field [2,43-48], several have shown a low distribution of *E. coli* strains in yoghurt [45-48]. However, Rahimi et al. (2011) [47] failed to detection *E. coli* strains in yogurt. Unfortunately, there were no published data on the presence of *E. coli* strains in kashk and doogh.

One possible explanation for the low prevalence of *E. coli* strains in yoghurt, doogh and kashk is that all of these dairy samples are fermented. In addition, acidic pH and high temperature during their process cause to low distribution of *E. coli* strains. Therefore, majority of contamination rates in the studied dairy samples are occurred due to the cross contamination. In keeping with this, adequate heat and time were not performed in the traditionally production of dairy. Also, native people have no access to healthy water in many sites of Iran. High potential of polluted water in dairy contamination has been reported previously [49].

The high incidence of O157 (26%) and O26 (12%) serogroups in dairy products of our study have been also reported previously by Momtaz et al. (2012) [2], Caro et al. (2006) [50], Madic et al. (2011) [51] and Pradel et al. (2008) [52]. It seems that majority of STEC strains isolated from dairy samples harbored O157 and O26 serogroups. We found statistically significant (*P* <0.05) differences between the incidence of O157 and O26 with other detected STEC serogroups. The most commonly detected serogroups in the study of Njage et al. (2012) [53] were O157, O111 and O113. Momtaz et al. (2012) [2] reported that O157, O145, O128, O121, O113, O111, O103, O91, O45, and O26 serogroups were detected in 14 (13.72%), 2 (1.96%), 4 (3.92%), 3 (2.94%), 3 (2.94%), 10 (9.8%), 6 (5.88%), 2 (1.96%), 6 (5.88%), and 18 (17.64%) dairy samples, respectively.

Another important finding relates to the distributions of several bacterial virulence factors in the dairy products of our study. We found statistically significant (*P* <0.05) differences between the incidence of *stx1* and *stx2* genes and between the EHEC and AEEC subtypes (*P* <0.05). The presence of multiple *stx1*, *eaeA*, and *ehly* genes was found in all of the EHEC strains (100%). Also, the presence of multiple *stx1* and *eaeA* genes, *stx2* and *eaeA* genes and finally *stx1*, *stx2* and *eaeA* genes were found in 45.16%, 32.25% and 22.58% of AEEC strains, respectively. Similar findings have been reported by Momtaz et al. (2012) [2], Mansouri-Najand and Khalili, (2007) [54] and Stephan et al. (2008) [55]. The same study found that out of the 77 *E. coli* isolates, 25 (32.46%) could be classified as Shiga-toxigenic based on PCR results (11, 3 and 11 isolates were positive for *stx*1, *stx*2, and both *stx*1 and *stx*2, respectively) [56]. Virpari et al. (2013) [57] showed that out of 80 *E. coli* isolates, 25 isolates (31.25%) were positive for *stx* genes, of which 7 (8.75%) isolates were positive for *stx*1 gene only, while 12 (15.00%) isolates were positive for *stx*2 gene and 5 (6.25%) isolates were positive for both *stx*1 and *stx*2, 7 isolates (8.75%) were positive for *eaeA* gene and all isolates were negative for *rfb* O157 gene.

We found statistically significant (*P* <0.05) differences between the incidence of genes encode resistance to tetracycline and trimethoprim. There were no significant differences between the incidence of *tetA* and *tetB* and *cat1* and *cmlA* genes. We found statistically significant (*P* <0.05) differences between the incidence of antibiotic resistance against tetracycline and enrofloxacin and also penicillin and trimethoprim.

Bacterial resistance against chloramphenicol and nitrofurantoin 30% and 22%, respectively. Chloramphenicol and nitrofurantoin are banned antibiotics and the high antibiotic resistances to these drugs detected in our study indicate that irregular and unauthorized use of them may have occurred in Iran. High bacterial resistances against chloramphenicol, nitrofurantoin, tetracycline, ampicillin and gentamicin have been reported previously [2,4,19].

## Conclusions

In conclusion, we identified a large number of serogroups, virulence factors and antibiotic resistance genes and resistance to more than one antibiotic in the *E. coli* strains isolated from yoghurt, doogh and kashk. Our data indicate that O157 and non-O157 STEC strains are predominant in Iranian dairy samples. Our data revealed that the O157 serogroup, the *stx1*, *stx2*, *eaeA* and *ehly* putative virulence genes, the *tetA*, *tetB*, *blaSHV* and *CITM* antibiotic resistance genes, and resistance to tetracycline, penicillin, ampicillin and streptomycin were the most commonly detected characteristics of the *E. coli* strains isolated from yoghurt, doogh and kashk. Careful hygienic supervision on the processing and packaging of dairy products should be performed to reduce load of *E. coli* contamination. Adequate heat and time should also be performed in the traditionally production of dairy.

## Abbreviations

E. coli: *Escherichia coli*; STEC: Shiga toxin producing *Escherichia coli*; EHEC: Enterohemolysin or Enterohaemorrhagic *Escherichia coli*; PCR: Polymerase chain reaction; Stx: Shiga Toxin; Eae: Intimin; Ehly: Hemolysin; Cnf1: Cytotoxic Necrotizing Factor; HUS: Hemolytic Uremic Syndrome; CDT: Cytolethal Distending Toxin; SPSS: Statistical Package for the Social Sciences.

## Competing interests

The authors declare that they have no competing interests.

## Authors’ contributions

FSD carried out the molecular genetic studies, participated in the primers sequence alignment, design the study and writing and drafted the manuscript. FY and JM carried out the sampling and culture method. YV participated in the statistical analysis. All authors read and approved the final manuscript.
